# Additive manufacturing technique-designed metallic porous implants for clinical application in orthopedics

**DOI:** 10.1039/c8ra04815k

**Published:** 2018-07-16

**Authors:** Chaohua Gao, Chenyu Wang, Hui Jin, Zhonghan Wang, Zuhao Li, Chenyu Shi, Yi Leng, Fan Yang, He Liu, Jincheng Wang

**Affiliations:** Department of Orthopedics, The Second Hospital of Jilin University Changchun 130041 P. R. China heliu@ciac.ac.cn jinchengwang@hotmail.com; Hallym University 1 Hallymdaehak-gil Chuncheon Gangwon-do 200-702 Korea; School of Nursing, Jilin University Changchun 130041 P. R. China

## Abstract

Traditional metallic scaffold prostheses, as vastly applied implants in clinical orthopedic operations, have achieved great success in rebuilding limb function. However, mismatch of bone defects and additional coating requirements limit the long-term survival of traditional prostheses. Recently, additive manufacturing (AM) has opened up unprecedented possibilities for producing complicated structures in prosthesis shapes and microporous surface designs of customized prostheses, which can solve the drawback of traditional prostheses mentioned above. This review presents the most commonly used metallic additive manufacturing techniques, the microporous structure design of metallic scaffolds, and novel applications of customized prostheses in the orthopedic field. Challenges and future perspectives on AM fabricated scaffolds are also summarized.

## Abstract 2

The bone defects can be investigated according to 3D data from computed tomography and magnetic resonance imaging, and then treated by CAD software for model and topology optimization to fabricate the customized implants.

## Graphical abstract

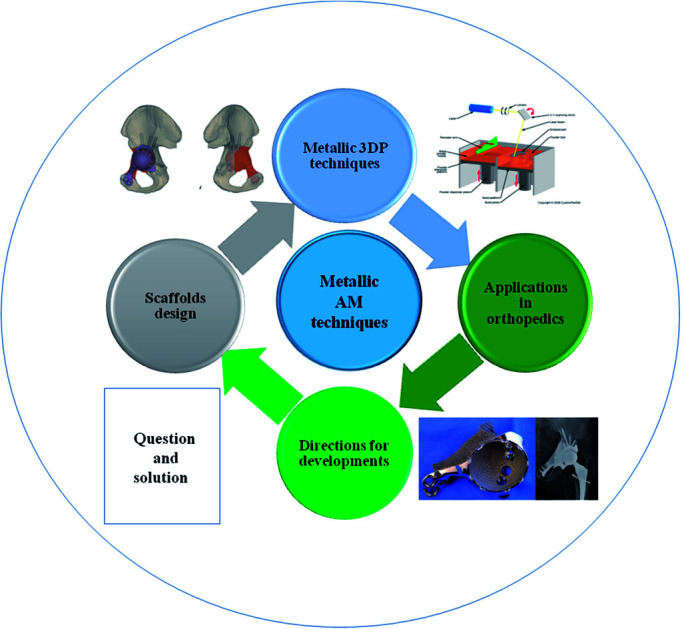

## 1. Introduction

Additive Manufacturing (AM) is a layer-by-layer fabrication method which can fabricate three-dimensional (3D) parts through computer-aided design (CAD).^1,2^ This technique provides advantages over traditional techniques as it can fabricate unique and complex structures in short term. It is said that AM, known as 3D printing (3DP), will be the next global industrial and technological revolution.^3,4^ Nowadays, AM technology has been widely used in many fields, such as the motor vehicle, aviation and medical fields.^5,6^ The medical application, especially in orthopedics, of the AM technique is a hot spot that account for 16.4% in the AM market.^7^ Based on the fabrication principle, AM techniques for metallic part fabrication are generally classified into five categories: selective laser melting (SLM), selective electron beam melting (SEBM), direct energy deposition (DED), binder jetting and sheet lamination. Each technology has its own advantages and limitations, so it is necessary to compare these features to meet different requirements.

The available materials of 3D printing include ceramics, polymers, metals, *etc.*^8,9^ Metals and alloys are widely used in orthopedic prosthesis fabrication, due to fine biocompatibility, superior mechanical strength and excellent corrosion resistance.^10–12^ However, the mismatch of elastic modulus between metals (110 GPa) and natural bones (4–20 GPa) may result in stress shielding, which can lead to the loosening and eventual failure of implants.^13–16^ In order to reduce stress shielding, an effective method is to use porous metallic structures through adjusting pore feature.^17^ In addition, porous structure can offer space for bone in-growth without the aid of additional coating. The ideal design of metallic scaffold should compromise three characteristics, *e.g.* appropriate surface roughness, high permeability and suitable mechanical properties to match host bone.^18,19^ Although high permeability may promote implant fixation, the mechanical properties are reduced with the increasing permeability.^20,21^ Therefore, it is necessary to trade-off the relevance of permeability (porosity) and mechanical properties (stiffness). In addition, the optimal design for pore sizes and distribution is also important indexes to implant osseointegration.

Over the last decades, conventional prostheses have achieved great success in orthopedics. However, there are many problems of conventional prosthesis remain to be solved, such as failing to fulfill bone defect perfectly and poor implant osseointegration, which may cause implant failure and even implant revision.^22,23^ The customized implants can be fabricated to fill bone defect according to 3D data from computed tomography (CT) and magnetic resonance imaging (MRI), and then treated by CAD software for model and topology optimization.^24^ Herein, 3DP implants show huge potential in orthopedics field, and 3DP technique provides new possibility for fabricating complicated implants with customized design.^25^

**Fig. 1:**
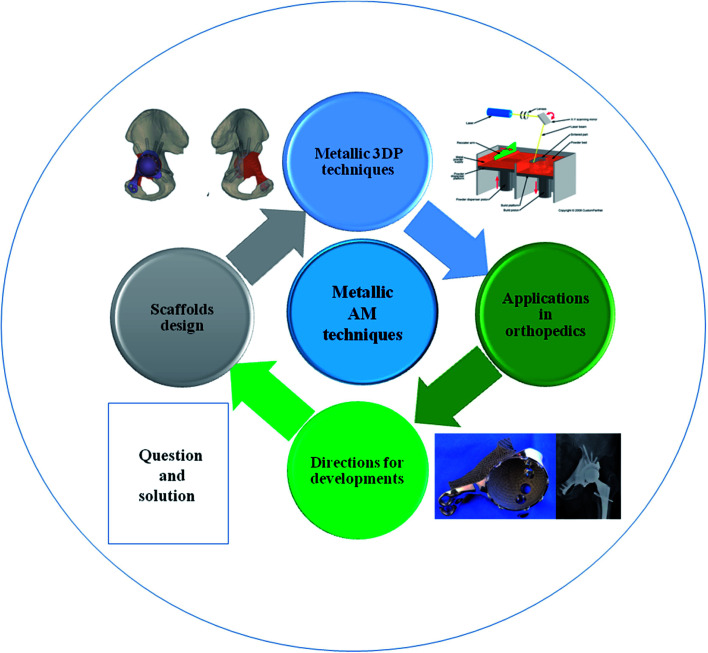
Scheme of metallic 3D printing techniques and design of metallic prostheses with regards to clinical applications in orthopedics.

This review summarizes various metallic 3DP techniques, the novel design of metallic scaffolds as well as clinical applications of customized prostheses in orthopedics (Fig. 1). The working principles, advantages and disadvantages of each technology will be described and compared. Then, the optimal design of surface roughness, the relationship between porosity and stiffness, pore size and distribution will be discussed in detail. Finally, the novel investigations of 3DP orthopedic implants as well as future perspectives will be also presented.

## 2. Techniques of metallic additive manufacturing

**Fig. 2:**
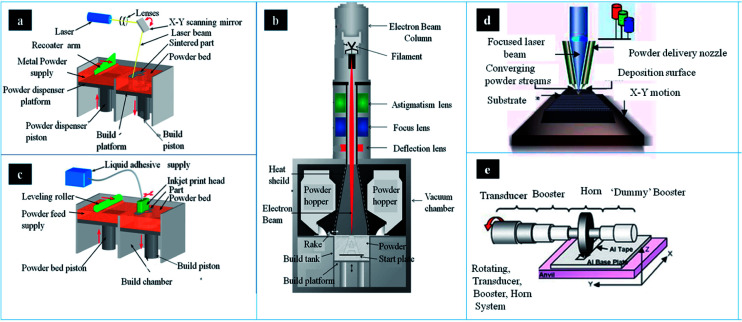
The schematic of main components and working process in different metallic AM systems: (a) SLM, (b) binder jetting (c) EBM system (d) LENS system and (e) UAM welding system. This figure has been reproduced from ref. 26–30 with permission from http://www.custompartnet.com and http://www.arcam.com.

Nowadays, there are several metallic 3DP systems in the market, including SLM, SEBM, DED, binder jetting and sheet lamination (Fig. 2). In this section, the process principle, parameters, advantages and disadvantages are described and compared.

### 2.1. Selective laser melting (SLM)

There are several devices in the SLM system, including laser source, powder containers, delivering and layering apparatus, build platform and computer systems (Fig. 2a).^31^ The first step is creating a 3D model through CAD software, followed by mathematically sliced thin layers. These layers are transferred onto the special device in order to fabricate 3D model layer by layer. Afterwards, metallic powder is deposited onto the build substrate with a thickness equal to the sliced thin layer. According to CAD model, the powder bed is scanned and processed repeatedly until the build is completed.^32^ In this process, the operating temperature is below 200 °C. Temperature monitoring system can provide information of the melt pool, which may improve part quality though uniform temperature distribution. There are two types of temperature monitoring system: pyrometers and thermocouples. Pyrometer is composed of photodiodes and digital cameras (*e.g.* complementary metal oxide semiconductor (CMOS), charged-coupled device (CCD)). Lott *et al.* used a CMOS camera to analyze images of melt pool size for control laser output powder.^33^ Thermocouples are also used in temperature monitoring in this process, which is low freedom than pyrometer owing to its contact measurement. This equipment cannot only record residual stresses and but also control the powder effective conductivity and energy absorption.

In order to prevent oxidation, purified argon or nitrogen can be introduced into the system.^34^ The metallic powder is loaded in the tank, then delivered onto the build platform, and recoated into layers, usually 30–100 μm.^35^ The morphology and granulometry of powders are important to flat, uniform layer. In addition, the property of powder can significantly affect layer thickness and surface roughness. Therefore, the feature of powder is crucial to the density and quality of the parts. In addition, metallic powders should possess small size distribution range and good sphericity.^36^ The optimal shape in SLM is spherical shaped powder, which is usually fabricated by atomization. However, only limited powders are prepared by this method. Therefore, it is needed to produce less spherical power by other method. Ball milling is an alternative method to prepare near-spherical powders.^37^ However, the long-time of ball milling can lead to irregular shape of power particles. The irregular shape may increase porosity of products, thereby leading to reduced quality of final parts.^38^ It was found that short time may be better in preparing near-optimal power, especially in titanium matrix alloy and oxide dispersion strengthened steel.^39–43^

The geometry of scanning can be designed in different ways. There is usually composed of straight and parallel lines, which may form circular or spiral coverage. Moreover, the direction can be altered inside a single layer or between consecutive layers. Therefore, the design of pattern can influence the quality of parts. Various laser parameters can be altered to make up a specific metallic model.^15^ The temperature of powers depends on laser energy density. A higher energy density would increase the amount of melting, therefore increasing final density. Therefore, a minimum critical density is able to be the optimal diversity to fabricate parts with maximum density.^44–47^ There are different methods to increase the laser energy density, such as decreasing layer thickness or scan spacing or scan speed, and increasing laser power. In addition, layer thickness exerts an influence on production time in SLM process. The connection between layers can take place when processed layers are re-melted. If layer thickness is reduced, the production time is increased.

SLM is an attractive method to produce complex shapes due to its several advantages, such as high mechanical property, high accuracy (50–200 μm), high material utilization, and net shape ability.^48,49^ However, it also has several disadvantages. In order to improve accuracy, the thinner layer thickness is needed, which can take a long time. It is difficult to be used in large-scale manufacturing. Residual stress is the common phenomenon that may cause interlayer de-bonding and stress cracking. These cracks may decrease the mechanical properties and dimensional accuracy.^50^ The heating/cooling rate is high in SLM process, which leads to high residual stress in final products. Hence, preheating build substrate can reduce the residual stress.^51^ In addition, the level of residual stress can also be reduced by post weld heat treatment. Balling effect is undesirable metallurgical process during SLM.^52^ In this process, laser energy can help the formation of liquid track. With the decrease of surface energy, the liquid track can be changed from cylindrical shape into spherical shape. There are mainly two drawbacks. On one hand, the formation of interline bonding was poor due to this effect. On the other hand, it is difficult to deposit metallic powder on the former layer uniformly, which may lead to delamination and porosity.^31^ Thus, it is important to minimize the balling effect, such as increasing laser power or reducing scan speed.

### 2.2. Selective electron beam melting (SEBM)

Selective electron beam melting (SEBM), as a powder bed fusion technique in AM, was developed by Arcam AB Corporation. There are several component parts in this system, such as power hoppers, rake, energy source, as well as build platform (Fig. 2c). The powder is supplied from two hoppers in the build chamber, and controlled by a moving rake. Then a powder layer can be spread over the build platform. In SEBM, the electron beam is used as the energy source. Electrons can be accelerated to a high velocity under 60 kV. Then, with the help of electromagnetic lenses, these electrons are focused into energy beam.^53^ The electron beam pre-heats powder layer, and followed by melting the powder layer according to CAD file. The outer boundary, defined as contouring, of the part can be fabricated firstly. Then the powder is melted in the contour to finish one layer. This process can operate until the part is completed.^54^ Infrared (IR) camera is an important tool that can be used in automatic feedback control system for defect detection, which can stop working process when porosity has certain level. All data would be achieved and optimized by this system in order to attain temperature automatically. Mireles *et al.* also deposited IR thermography in EBM system for *in situ* defect detection. These image data can offer information of defects geometry and be used in *in situ* correction through re-melt defect.^55^

The powder properties are important to process stability and products properties. High flowability of powder is required in this process. In general, spherical gas atomised powders would be the first choice. The aimed powder size is between 40 and 105 μm. In order to maintain process stability, powders with larger mean diameter are better than smaller ones. Therefore, powders would be produced by plasma rotation electrode. The aimed thickness of the powder layer is varying between 50 and 150 μm.^56^ Owing to larger powders size, layer thickness and beam diameter, the surface roughness of SEBM parts is much higher than that of SLM. In general, the values of SLM and SEBM are *R*_a_ = 11 μm and *R*_a_ = 25–35 μm respectively.^57^ In addition, smoke events may take place due to the repulsion of powders with charge, which can result in process instabilities.^58^ Therefore, it is necessary to pre-heat the start plate in order to sinter the powders a bit. Sintering the powder is essential to increase the electric conductivity, which can avoid process instabilities. In the end of SEBM, the powder can be recycled and reused under proper conditions.^59^ In this process, a part of molten powders will stick together, which needs to be removed by sieving. The small satellites can be gradually removed in each process. Thus, it can improve the power properties and process stability.

SEBM is only restricted to metals due to electric conductivity. Owing to inherent features of electron beams, it is needed to operate under high vacuum (10^−4^–10^−5^ mbar). The reaction of powders with air can be avoided in vacuum, which can maintain the quality of parts.^17^ It was reported that SEBM is able to be the preferred method in producing Ti–6Al–4V, owing to the clean environment.^60^ The energy density of beam is above 100 kW cm^−2^, which melts metallic powders fully. In addition, the scanning speed of SEBM can reach to 105 m s^−1^. Thus, SEBM is faster in producing parts with high quality than SLM. Component precision is an important topic in AM. Unlike SLM process, SEBM is performed at higher temperature. In addition, there is a decrease in cooling rate and temperature gradient.^61^ The residual stresses and stress-relief annealing of SEBM is lower than SLM.^62^ It was reported that component precision is ∼100 μm. Moreover, the solidification cracking can be minimized in EBM, owing to low cooling rates.

### 2.3. Direct energy deposition (DED)

DED process can be used in depositing feedstock into melt pool by focused energy. The feedstock includes powder or wire. The energy source may be a laser, arc or e-beam. Laser engineered net shaping (LENS) is the most common form of DED that has been patented, which was developed by Sandia National Laboratory, and then commercialised by Optomec Inc^63^ (Fig. 2d). It is an important AM technology that belongs to the direct laser deposition techniques. This technique can not only be applied in the fabrication of near net-shaped product, but also used in surface modification. In this process, the molten pool is not surrounded by a powder bed.^64^

The powder hoppers should be filled with powders. In addition, powders can be fed through nozzles, and then delivered to the central point of laser. The power laser of LENS is up to 4 kW. In LENS, it is necessary to check the feed rate of powders constantly. Once powder flow is obstructed, nozzle needs to be cleaned. The powder can be melted into a micro-melt pool, which can adhere to the substrate.^65^ The build substrate can be also positioned originally, while the laser system moves upward among each layer. This system can control speed, accuracy, and property by tailoring deposition parameters. Nowadays, there are many monitor and control systems applied in this process for higher quality. High speed camera and CCD camera can be applied in the collection of information through nozzle and powder delivery rate. Using these information, design of experiment (DOE) be used in the analysis of the nozzle dimension and powder size along according to changes of distance and gas flow.^66,67^ Smurov *et al.* positioned high-speed camera on the nozzle head for monitor the powder delivery rate.^68^ Bi *et al.* designed closed-loop controller using IR pyrometer, which can collect information of melt pool temperature. This system can not only send monitored temperature feedback, but also tolerate laser powder density.^69^ Hu *et al.* developed a real-time sensing and control system that monitor and control the rate of powder delivery using IR high speed camera. The data of melt pool is collected by camera, and delivered into sensor to adjust laser powder, which can improve the quality of 3D part.^70^ In order to monitor layer height, Iravani-Tabrizipour *et al.* developed CCD cameras and neural network algorithm in real-time.^71^ The closed build chamber can ensure laser safety. For active metals, the chamber needs to be filled with inert gas.^72^ The chamber is much larger than other systems. Thus, purge cycles can consume plenty of inert gas for decreasing oxygen partial pressure. There are several advantages in LENS compared to powder bed fusion, such as better cooling effect, re-fabricating capability.^65^ With the help of powder feeding gas coolant, the cooling rates of LENS is high in this process. In addition, it can produce parts with high mechanical property. However, this technique also has disadvantages, such as low fabrication efficiency and high roughness.

### 2.4. Binder jetting

In general, there are two materials in this process: metallic powder and binder material, which can create a bond with powders.^73^ The binder is in the form of liquid. A layer of powder (20–100 μm) is spread and then binder is deposited on the layer through CAD model. It can later consolidate the powder or infiltrate with other metal. The consolidation can produce uniform sample with single metal. While infiltration can achieve dense products using metal with lower melting temperature. In this process, the printer can deposit binder on metallic powder (Fig. 2b). Binder-metal sample, known as green body, can be removed from this system once the binder dries. In order to improve mechanical strength, the sample should be cured. The product is then needed to sinter the bound powder and burn off binder by heat treatment at 1100 °C for 24–36 hours. The sintered sample can achieve a density of 60%. It can infiltrate the liquid metal into the partially sintered scaffold by capillary action.

The print model of binder jetting technique includes uni-directional and bio-directional mode. Although bio-directional one is faster than uni-directional one, it was poor in quality surface due to dynamic shift errors. In order to minimize the binder accumulation and pump clogging, researchers developed a 45 degree print head rotation that can promote dynamic shift and attain accurate droplets on powder bed, which can improve surface finish and printing accuracy.^74^ There are several factors effect on part dimensions, including accuracy of droplet placement and of deposited layer thickness, reproducibility of droplet spread, and dimensional change in curing process. The printing orientation can perform effect on dimensional accuracy and mechanical properties of 3DP scaffold. Farzadi *et al.* reported that the relation of printing orientation (*i.e.* longitudinal direction) and printing head movement can lead to better dimensional accuracy and mechanical properties.^75^

The shrinkage is a difficult problem in sintering process. The error in accuracy of part is mainly caused by shrinkage during cooling and solidification due to internal stresses. In this period, internal stresses may cause cracking and delamination of parts.^76^ It was reported that geometrical accuracy is 350–500 μm. In order to match uniform shrinkage, it should be designed in distortion of the geometry before. However, the designed distortion cannot adapt to the shrinkage. Thus, the quality of sample may be not very well in consolidation.^77^ Moreover, the sample should be sintered until the part becomes the final geometry. Surface finish of binder jetting accord with PBF processes. The surface finish of sample after annealing is at 15 μm [*R*_a_], and post-processing is only at 1.25 μm [*R*_a_]. There is no residual stress in the sample owing to no heating in the building process. It may result in porosity by sintering, leading to the decrease in mechanical properties.^78^ Therefore, the part is not suitable for high mechanical strength. In addition, there are many advantages in this technique. Binder jetting is able to be one of the most cost-effective processes in AM.^79^ The printing speed of binder jetting is fast relatively. In addition, the speed can be accelerated by raising print head holes. It can regulate mechanical properties by altering the powder–binder ratio. There are several topics in the future, such as geometrical accuracy, better infiltration materials and binder burn off.

### 2.5. Sheet lamination

Sheet lamination is a stacking process of metal sheets that is cut from 3D sample. The metal sheets cannot only be cut in order, but also be further cut into specified geometry. Then, these metal sheets are bonded metallurgically or joined adhesively. There are many techniques used in the process, such as ultrasonic consolidation, laser welding, diffusion bonding, resistance welding and brazing.^80–82^ In Sheet lamination process, the ultrasonic additive manufacturing (UAM) is one of most promising techniques (Fig. 2e). The UAM is an ultrasonic seam welding technique, by which the metal sheets (layer thickness: ∼100 μm) may be joined together.^83^ The UAM process can deposit metal tape onto other tape layers by ultrasonic welding. Then trim the edge of layers in order to meet specified geometry. The two layers are joined until the product is completed. At last, machining or milling is required to produce channels, holes and so on. It was found that the interfaces possess good bonding, which has a recrystallisation grain texture. In addition, the grains at the interface were very stable. The interface temperature, in localized region, can increase to 380 °C in the process.^84^ However, the temperature of whole part is still low.

Laminated object manufacturing (LOM) is another sheet lamination technique, which can perform heat or pressure on the metal sheet through a heated cylinder rolling. The vertical surface roughness of LOM part is the vital quality characteristic, which is a hot spot in this field. It was found that higher vertical surface quality can result in lower post-processing time, less finishing and higher process optimization.^85^ In order to optimize LOM process parameters, J. Kechagias *et al.* developed a model through a feed-forward back propagation neural network (FFBP-NN). This model can be used in the prediction and selection of optimal process parameters.^86^

Sheet lamination process has some advantages, including ease of making large-scale parts, good surface finish, low geometric distortion and low costs. It can also build a layer with fine accuracy and resolution. However, it also has some limitations. It is difficult to reach geometric accuracy, in the *Z*-direction, owing to swelling effects.^87^ Due to no mechanical support, it is impossible to produce complex overhangs.^88^ In addition, anisotropic properties are common in the final parts due to different techniques. The joined parts may be poor in shear and tensile loading conditions. At last, the process may be also limited by the tool paths in machining operation.

Nowadays, metallic AM technology would be the most promising technique in medical applications. There are mainly five techniques based on own fabrication principle. According to advantages and limitations, it is useful to choose appropriate technique (Table 1). In addition, parameter of selected metallic AM technology can also be optimized to enhance part quality. Thus, the metallic implants fabricated by AM show huge potential in orthopedic field.

**Table 1:** The techniques of metallic additive manufacturing and the process principle, parameters, advantages and disadvantages

Techniques	Process principle	Parameters	Advantages	Disadvantages
SLM	A thin layer of powders can be selectively melt using laser beam layer by layer.	Layer thickness: 30–100 μm	Complex shapes manufacturing, high mechanical properties, high accuracy, high material utilization, and net shape ability.	Time-consuming (difficult in large-scale manufacturing), high residual stress.
		Smallest feature: 50–200 μm		
SEBM	A thin layer of powders can be selectively melt using electron beam, which is repeated for each layer.	Layer thickness: 50–150 μm	Complex shapes manufacturing, low residual stress, high mechanical properties, high accuracy, time-saving.	High surface roughness, smoke events.
		Smallest feature: ∼100 μm		
LENS	Powders are delivered to the laser beam, melted and deposited onto a substrate. This system is controlled by the CAD models until the part is produced.	This system can control speed, accuracy, and property by tailoring deposition parameters.	Complex shapes manufacturing, net shape ability, high mechanical properties, high accuracy.	High surface roughness, time-consuming
Binder jetting	A layer of powder is spread and then binder is deposited on the layer through CAD model. This process is repeated until part is produced.	Layer thickness: 20–100 μm	Cost-effective, high efficiency, no residual stress.	Shrinkage, poor quality, surface finish, low mechanical properties.
		Smallest feature: 350–500 μm		
UAM	This process can join metal sheets based on CAD design using ultrasonic energy. Then excess part is trimmed out using CNC machine.	Layer thickness: ∼100 μm	Large-scale parts, good surface finish, low geometric distortion and low costs.	Poor geometric accuracy, poor in complex overhangs, poor in shear and tensile loading conditions.

## 3. Requirements for the design of metallic scaffold

Bone has a hierarchical structure, including cortical bone and cancellous bone. The cortical bone is almost solid and only 3–5% spaces are left for osteocytes, blood vessel and so on. However, there are larger spaces in cancellous bone filled with bone marrow, which is made of porous network with a porosity of 50–90%. The elastic moduli of cortical bone is 3–30 GPa, while cancellous bone is only 0.02–2 GPa.^89^ In order to decrease stress shielding of metallic scaffold, porous design of inner structure would be an alternative option. In addition, porous structure can also provide open space for bone regeneration, which can enhance long-term stability of implants.^90^ Nowadays, numerous studies focused on the design of scaffold. The ideal design of metallic scaffold mainly has three characteristics, including proper surface roughness, enough permeability and appropriate mechanical properties. Although high permeability may benefit implant fixation, the mechanical properties are reduced with the increasing permeability. Therefore, it is needed to maintain trade-off between permeability (porosity) and mechanical properties (stiffness). Recently, the optimal design for pore sizes and distribution is still controversial. In this section, we will discuss the recent investigations of surface roughness, the relationship between porosity and stiffness, pore size and distribution of metallic microporous scaffold.

### 3.1. Surface treatment

Although metals and alloys are excellent materials in implants, there are also many challenges in surface characteristics. One of challenges is bio-inert that is difficult to interact with host bone. This property may lead to fibrotic lining or scar tissue between bone–implant interface, which may decrease implant fixation.^91^ The modification of implant is important to the success of metallic prosthesis. In general, metallic prostheses have smooth surfaces with low wettability that is not suitable for cell adhesion. The relationship between surface roughness and cellular proliferation or differentiation is also useful to orthopedic implants. It was found that there is increasing cellular proliferation with the increasing of surface roughness from 0.16 μm to 2.19 μm.^92,93^ While other researchers also reported that enhanced osteoblast proliferation was observed on smooth surface, and a higher differentiation on rough Ti surface.^94^ Many researchers believed that surface treatment is necessary. Li *et al.* treated Ti6Al4V scaffold with heat treatment by different temperatures (800 °C, 950 °C and 1000 °C), and investigated their effect on mechanical properties, roughness and bone ingrowth capacity.^95^ It was reported that the mechanical properties and roughness were improved with the increase of temperature. Compared with untreated scaffold and treated scaffold at 800 °C, the scaffold treated at 950 °C and 1000 °C can attain higher cellular proliferation and better osseointegration. However, other researchers also thought that parts can be directly used without surface treatment.^96,97^ With the increasing of surface roughness, the implant with *R*_a_ below 24.9 can enhance the proliferation and differentiation of osteoblasts. However, surface roughness has a negative effect on the osteoblasts when *R*_a_ exceeds 56.9 μm.^98^

Surface modification of 3D scaffold is more difficult than that of solid material, and only limited techniques can be used in the process. Microarc oxidation (MAO) is an electrochemical technique for surface modification, which can form microporous oxide coating on metals with complex structure.^99,100^ Xiu *et al.* developed Ti6Al4V scaffold with uniform layer of microporous TiO_2_ as well as calcium-phosphate using MAO technique.^101^ It was found that bone in-growth was only at the periphery of untreated scaffold, while osteogenesis was in suit on the whole surface of MAO-treated scaffold. Moreover, the structure of microporous TiO_2_ can also provide higher implant fixation through bone/implant interlocking. HA coating is a common method for surface modification owing to its architecture and composition close to bone tissue. However, it is difficult to attain uniform HA coating on the inner surface of metallic scaffold. It was found that pDA coating can enhance HA formation on scaffold.^102,103^ Li *et al.* performed pDA-assisted HA coating onto the inner surface of Ti6Al4V scaffolds produced by EBM, and found that there was higher cellular attachment and proliferation, and even improved osseointegration and osteogenesis in treated group.^104^

### 3.2. Porosity and stiffness

Bone in-growth in porous scaffold mainly depends on recruitment of BMSCs from surrounding tissues.^105^ The permeability of scaffold is an important characteristic, which depends on gradient pressure to push liquid. Higher permeability can benefit the transportation of cells, nutrients and waste through the scaffold. Therefore, it can improve the osteoconductive potential of porous scaffold.^106,107^ Permeability modification can be realized by the change of porosity, and further influence bone in-growth and implant fixation. Moreover, bone formation was deeper and larger in scaffold with higher porosity. Numerous researches have been focused on this topic. In general, porosity of scaffolds should be more than 40%.^108^ In scaffold with porosity of less than 70%, there was better bone in-growth with higher porosity than lower porosity group.^109^ Similarly, Cheng *et al.* reported that better osteoblast differentiation in human trabecular structure with higher porosity (70%) than lower porosity (15% and 37.9%).^110^ In addition, scaffold with over 80% average porosity showed excellent implant fixation.^111^ Other researchers also demonstrated that scaffold with porosity similar to human trabecular bone (70–90%) showed better bone in-growth.^112^

The balance between mechanical properties and porosity is key consideration for implant design. The mechanical strength may be reduced by increasing porosity, leading to improved permeability, and the implant should match the stiffness of bone in order to avoid bone resorption.^113^ Murr *et al.* fabricated different porous Ti6Al4V implants by EBM. It was found that the elastic modulus could increase from 0.58 to 3.03 GPa, when the porosity decreased from 88% to 59%.^114^ Pattanayak *et al.* manufactured Ti scaffolds by SLM based on cancellous bone. When the porosity decreased from 75% to 55%, the compressive strength increased from 35 MPa to 120 MPa.^15^ The titanium scaffold with porosity of 66% had elastic modulus of 2.5 GPa, which is similar to natural bone.^115^ It was found that stretch-dominated scaffold with high porosity has strong mechanical strength but low modulus.^116^ Hence, the design of this scaffold is needed in the future.

**Fig. 3:**
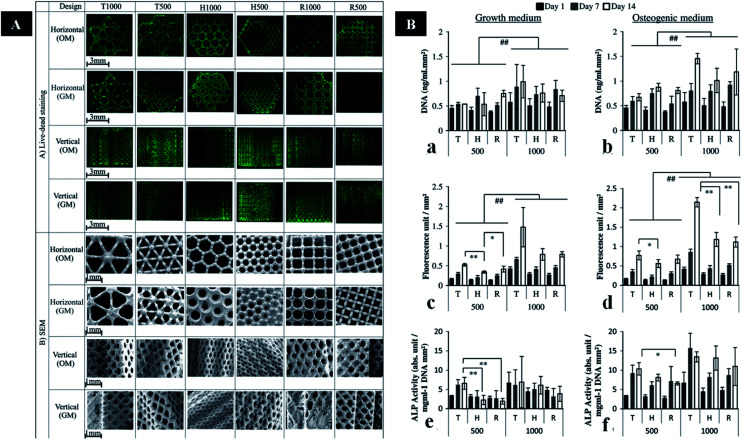
(A) Images of live/dead staining and SEM images in the horizontal and vertical planes of cells on six porous Ti6Al4V scaffold designs. (B) DNA assay, and metabolic and ALP activity of cells on different porous Ti6Al4V scaffold designs cultured in GM or OM. This figure has been reproduced from ref. 117 with permission from Elsevier.

The pore shape and sizes can also affect mechanical properties of scaffolds to some extent. Van Bael *et al.* fabricated scaffolds including different pore sizes (500 and 1000 μm) with three shapes (triangular, hexagonal, and square). The scaffolds with hexagonal pores possess the highest compressive strength. The hexagonal pores with 500 μm are close to cortical bone. While the mechanical properties of triangular and square scaffolds are similar to cancellous bone (Fig. 3).^117^ The porous scaffold can be optimized in order to maintain trade-off between strength and porosity. Thus, the balance of mechanical property and porosity using different AM techniques should be deeply investigated in the future.

### 3.3. Pore size and distribution

Many studies investigated the optimal pore sizes of metallic microporous scaffold for bone in-growth. It was suggested that 100–400 μm would be the suitable range of pore sizes.^118^ While, other studies found that new bone can be formed in microporous scaffold with small pore size (50 μm and even less than 10 μm).^105,119^ However, there are high proportions of occlusion in small pores, and it is difficult to produce small pores with fine definition using AM technique.^120^ Kuboki *et al.* reported that pore size with 300–400 μm can favour bone regeneration and formation.^121^ In addition, bone regeneration could be increased with pore size. The rate of bone in-growth in 600 μm and 900 μm pores was faster than 300 μm pores.^122^ This phenomenon can be explained by vascularization, which can accompany with bone regeneration. Bone is also a natural scaffold composed by highly vascularized tissue.^123,124^ Pore structure of scaffolds can significantly affect the vascularization *in vivo*.^125^ The nutrition and oxygen is difficult to diffuse into scaffolds with small pore size, which can result in the formation of necrotic core. It was reported that vascularization may increase with the increasing pore size.^126,127^ However, there is no significant difference in vascularization with pore size more than 400 μm.^128^ Thus, pore size should exceed 400 μm at least. In addition, it is found that 50–800 μm is the recommended pore size.^111,129^ Therefore, we suggested that 400–800 μm would be the optimal range of pore sizes according to many factors. However, further studies should focus on the optimal pore sizes for both bone in-growth and vascularization.

**Fig. 4:**
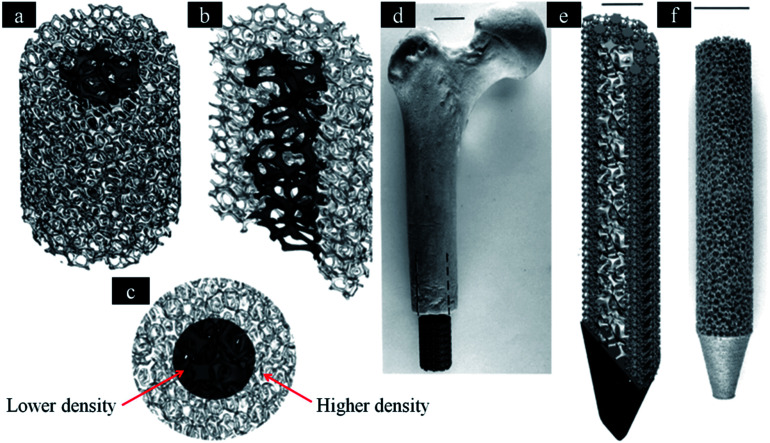
Software (CAD) models incorporating inner and outer foam elements. (a) Three-dimensional, (b) three-dimensional half-section and (c) end views, respectively. (d) Femoral prototype with mesh elements. (e) The inner foam and outer mesh elements for a femoral prototype (half-section view). (f) SEBM-fabricated femoral implant with porous structure similar to (a). This figure has been reproduced from ref. 132 with permission from The Royal Society.

This scaffold can rebuild different types of bone tissue in different sites. Hence, the design of scaffolds should possess varying porosities in different regions. It is necessary to produce scaffolds with hierarchical structures, which is similar to natural bone tissue. Thus, the implant could possess properties similar to host bone. On one hand, gradient scaffolds can simulate bone in-growth.^29^ On the other hand, the gradient structure can also minimize stress shielding effect. Rumpler *et al.* reported that cell growth in small pores (−500 μm) is better than large pores.^130^ However, large pores (−1000 μm) can promote the transportation of cells, nutrient substrate and metabolic waste. Thus, Van Bael *et al.* believed that graded lattice would be the optimal design.^131^ It is composed of small pore sizes in the inner region and large pore sizes in the outer region. The design can benefit transportation of nutrients and oxygen into the implant. In addition, larger pores can also avoid occlusion in the interface of bone and implant, which can help bone in-growth. While Murr *et al.* produced implants with complex functional cellular structures using metal EBM technique^132^ (Fig. 4). It is necessary to determine the optimal design of hierarchical structures for bone in-growth in the future. Conventional scaffolds are fabricated by randomly shaped pores in different pore sizes. AM techniques can be used in producing implant, which have different pore sizes within one sample.^133,134^ In addition, metallic scaffolds can be designed to imitate the structure of natural bone by AM technology. These techniques can fabricate optimal graded structures that possess mechanical strength and bone regeneration.^110^ Hence, it is necessary to compare mechanical property and bone in-growth of scaffolds with graded structures fabricated by these AM techniques. In addition, a reliable database of human bones should be established. This database includes related data of biological and mechanical properties based on different age, gender, race and locations. These data can be used in the design of scaffold.

## 4. Application of 3DP metallic implants in orthopedics

**Fig. 5:**
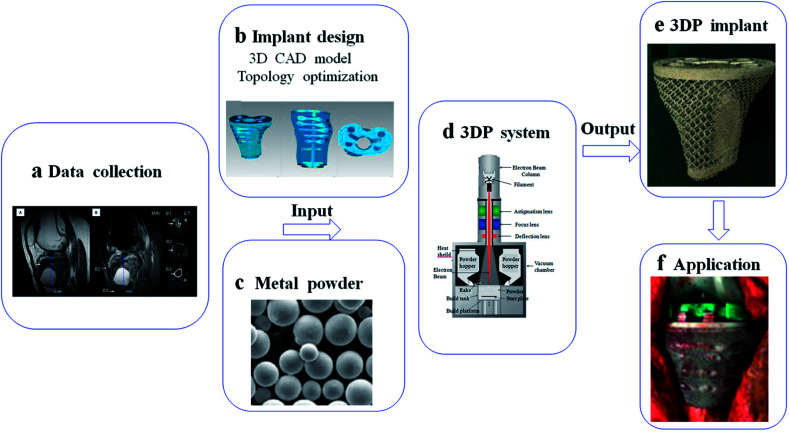
Schematic of basic steps for implant fabrication based on 3DP system: (a) data collection from CT and MRI; (b) implant design in computer for 3D CAD model and topology optimization; (c–e) implant fabrication in 3DP system using metal powder; (f) application in medical field. This figure has been reproduced from ref. 177 with permission from International Scientific Information.

AM technologies open up new possibilities for fabricating complicated implants with customized design.^135,136^ In this process, data collection for implant design can depend on CT and MRI, and then treated by CAD software for 3D model and topology optimization.^137^ With the help of 3D data, customized implant can be produced by 3DP system using metal powder. The patient-special implant has porous structure, which is perfectly fitted to the lesion and its boundary (Fig. 5). Here, we summarized several 3D-printed applications in clinical practice. In addition, the problem and direction of these implants will be mentioned.

### 4.1. Application of 3DP metallic implants in maxillofacial and oral field

Dental implants have achieved great interest over the last decade. Nowadays, one million people received these treatments per year around the world.^138,139^ However, dental implants also face certain limitations, especially when used in patient with low bone density. The implant osseointegration is poor in osteoporotic patient relatively, which can minimize the implant life-span. In addition, the traditional implant is non-porous that can lead to stress-shielding between implant and natural bone.^140^ This phenomenon can significantly decrease the implant fixation. Porous scaffold fabricated by AM can provide enough mechanical support and bone in-growth space for osteoporotic patient.^141^

Mechanical property is an important characteristic in the design of implant. In order to minimize the stress-shielding, many researchers have fabricated different dental implant using different AM techniques. Dental implants should have a rough surface, on which macroscopic grooves or porous surface can promote mechanical stabilization between implants and host bone. Tolochko *et al.* developed dental root implants with a compact core by SLM and porous shell by SLS. The porosity is 40–45%, and the pore size is 100–200 μm. This implant includes a large number of channels with 1 mm in depth and 1 mm in diameter.^142^ There are a series of variables in this process. The optimal parameters may be that hatching pitch is 0.4 mm, laser peak power is 1 kW, and scan speed is 6 mm s^−1^.^143^ The direct laser fabrication is an economic technique that can produce implants with shallow depressions and narrow intercommunicating crevices. It was reported that the elastic modulus of scaffold fabricated by EBM decreased with increased porosity.^144^ In addition, the mechanical properties can close to bone tissue by changing pore structure.^145^ It was found that the scaffold with different porosities ranging from 15%–70% can mimic trabecular bone. The trabecular structure is able to be suitable for dental implant.^146^ Traini *et al.* fabricated porous dental implants with graduated porosity by laser sintering. The modulus elastic of dense core was comparable to titanium implant, while porous part was similar to host bone.^147^ Other researchers fabricated porous Ti–Al6–V4 scaffold with 23–32% porosity by LENS. The modulus can match cortical bone.^148^ All in all, the mechanical supports should be enough between the superstructure crown of dental implants and its root. Thus, the implant with a solid core and porous shell would be a better choice.^141^ However, there is large stress concentration in the connection between the core and shell.^113^ Cook *et al.* suggested that post-sintering heat treatment can improve the fatigue strength of titanium by about 15%. Furthermore, graded structures can also minimize the stress concentration.^149^ Moreover, researchers also focused on the clinical outcomes, and found that excellent bone in-growth in porous scaffold fabricated by AM techniques. Mangano *et al.* fabricated porous titanium scaffolds by a laser sintering, and reported that 95% success in clinical observation followed by one year.^150^ This outcome can be explained by bone in-growth into porous structure, which can enhance implant osseointegration. All in all, metallic scaffold fabricated by AM can provide desired mechanical support and implant fixation. Nowadays, Zimmer is the only porous dental implant on the market. The porous structure of the implant can maintain a balance between stiffness and bone in-growth, which can attain desired implant fixation without implant failure.^151^ In order to improve the clinical outcomes, the feature of pores (shape, size, percentage and distribution) should be optimized in the future.^152^ Porous scaffold fabricated by AM would be one of most promising implants in the future, which can be widely used in dental field.

### 4.2. Application of 3DP metallic implants in spine

The traditional inter-body fusion cages can maintain disc height and provide comfortable environment to bone grafts for bony healing. Therefore, traditional implant is designed as cylindrical and hollow interior space for bone graft. However, excessive cage rigidity may lead to several complications, including the migration of the cage, stress-shielding and pseudoarthrodesis.^153,154^ In addition, there are individual differences in the disc height, which can prolong the operation time for selection of fusion cages. The mismatch between disc and cage may change the curvature of spine. Once the case combined with severe bone defect caused by bone tumor, traditional implant seemed helpless for further reconstruction.

**Fig. 6:**
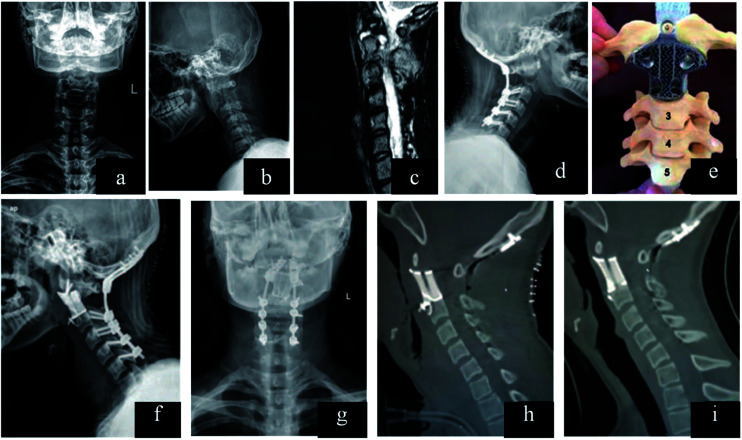
(a–c) Lesion and its boundary located in the preoperative X-ray and MRI. (d) After resection of the posterior C2 elements and fixation in the lateral X-ray. (e) Model showed that how the 3D vertebral body was inserted between C1 and C3 in the second surgery. (f and g) Anteroposterior and lateral X-ray after the second surgery. (h) Sagittal reconstruction after surgery and (i) at the 1 year follow-up demonstrating implant osseointegration without implant failure and local recurrence of the tumor. This figure has been reproduced from ref. 162 with permission from Wolters Kluwer.

Topology optimization can be applied in the design of cage with microstructure, which can attain desired stability, and reduced stress shielding. It may provide optimal distribution to meet the objective of sufficient stiffness with desired porosity.^155–157^ The strain energy may be absorbed by bone graft and formed bone inside the cage. Ti–Al6–V4 lumbar inter-body fusion cage fabricated by SLM can reproduce intricate microscopic structure, and the compressive modulus (2.97 ± 0.90 GPa) falls between the trabecular and cortical bone.^158^ Computationally designed lattices with tuned properties can be fabricated by 3D printing, which can provide optimal structure for bone tissue engineering.^159^ Researchers developed 3D prosthesis for posterior C1/C2 fusion, and the postoperative recovery was successful.^160^ The customized implant cannot only provide enough support, but also empty space for bone graft. 3D implant can be designed with fixation holes for pedicle screws, which was applied in reconstruction of T9 primary bone tumor.^161^ The designed implants can shorten the surgical time, minimizing further complex reconstruction. In addition, there are higher long-term stability and better recovery effect. Nowadays, Liu *et al.* designed 3D inter-body fusion device for upper cervical spine with C2 Ewing sarcoma (ES), which has been approved by Chinese Food and Drug Administration (CFDA). The microstructure of the implant can be optimized in order to keep a balance between porosity and implant fixation. There was excellent implant osseointegration without implant failure^162^ (Fig. 6). In the future, 3D implant with customized design would be an efficient device in spine surgery, which can shorten surgical time, minimize further reconstruction.

### 4.3. Application of 3DP metallic implants in joint

Over the last decades, conventional prosthesis has been applied in joint. However, these implants would not be a good choice to surgeon in severe bone defect around joint. The biomechanical balance of joint is important to every case. In addition, there are individual differences in the anatomy of joints. Therefore, traditional implant seemed helpless for large bone loss around joint. 3DP prosthesis with customized design would be an optimal candidate in these cases.

#### 4.3.1. Application of 3DP metallic implants in hip joint

**Fig. 7:**
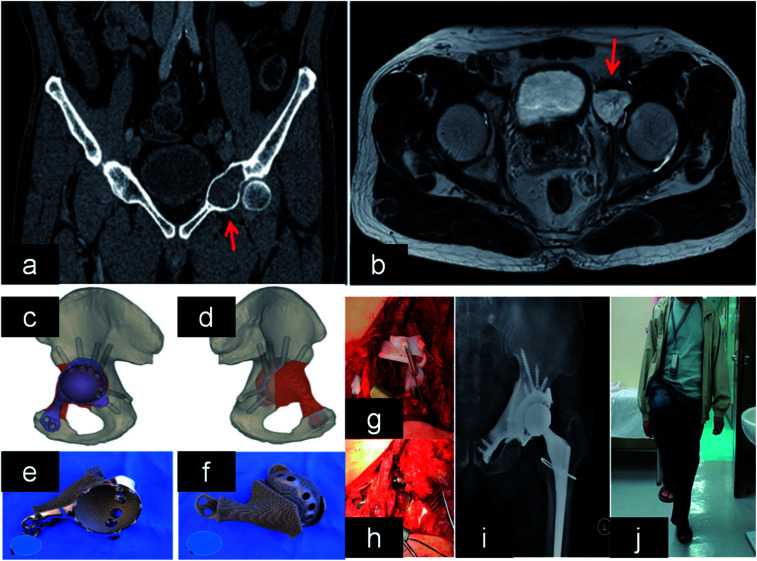
(a and b) Lesion and its boundary located in the MRI and CT. (c and d) The 3D pelvic model showed the implant reconstruction and screws fixation. (e) The outer view showed the acetabular cup with screws holes for fixation. (f) The back side showed the porous structure that was in contact to bone. (g and h) After tumor resection, the implant was fitted to the bone defect perfectly and stabilized with screws. (i and j) X-ray showed good implant alignment, and patient could stand with single leg at 10 months after the surgery. This figure has been reproduced from ref. 167 with permission from Taylor & Francis.

The reconstruction of large acetabular bone defects is still a difficult problem to surgeons. There are many treatment options for this condition, such as acetabular reconstruction cages, oversized hemispheric cups and so on. However, none of these treatments showed desired clinical outcomes. Nowadays, cage with porous structure has showed excellent osseointegration and implant fixation, which can be used in the reconstruction of acetabular bone defect.^163^ It was found that 95% integration with porous implant in 43 patients at 6 weeks.^164^ Moreover, there is no periprosthetic osteolysis around porous implant in many cases.^165,166^ Owing to the complex shape of acetabular bone defects, it is difficult to attain the match between cage and host bone. Initial stability on host bone can perform effect on long-term clinical outcome. Nowadays, these implants fabricated by AM can be used in these cases based on the shape of bone defect. The elastic modulus of porous implant was similar to human bone, which can minimize the stress shielding. In order to attain mechanical property, the pore size and porosity were adjusted to 0.72 mm and 70%. In addition, this design can promote bone in-growth, which favors implant biological fixation. Clinical results showed that the patient can walk a long distance without other aid^167^ (Fig. 7). It was reported that 24 patients were implanted with 3DP acetabular cage. Clinical outcomes reported that Harris hip scores (HSS) was improved, while complications was reduced.^168^ Therefore, 3DP acetabular implant would be an optimal candidate in the future. Although it has good short-term results, the long-term results are also necessary to be followed up.

Patients with periacetabular bone loss or pelvic discontinuity are challenge to orthopedic surgeon. These cages can provide support structure to maintain initial stability for cases with pelvic discontinuity. In addition, the hip center is able to be restored to anatomical position. Significantly, it was reported that porous implants can decrease the need for more than 50% bone contact.^169,170^ There were 25 cases of acetabular defects revised by 3DP cages. In addition, all cases had excellent implant fixation without mechanical failure or loosening followed by 4.4 years.^171^ Another study demonstrated that 3D acetabular triflange components can be applied in the repair of catastrophic bone loss.^172^ This design can promote bone in-growth, which favors implant biological fixation. Owing to limited bone contact and movement of bone fragments, it is difficult to attain reliable fixation of the acetabular component in pelvic discontinuity. In these cases, 3DP customized triflange acetabular component (CTAC) was implanted into bone defect precisely, and all implants showed reliable fixation in the ischium, pubis and ilium.^173^ Other researchers also implanted 3DP CTAC into pelvic discontinuity, and found that there were improved HHS score without complications.^174^ This technique is a reliable and promising option in these cases.

#### 4.3.2. Application of 3DP metallic implants in knee joint

**Fig. 8:**
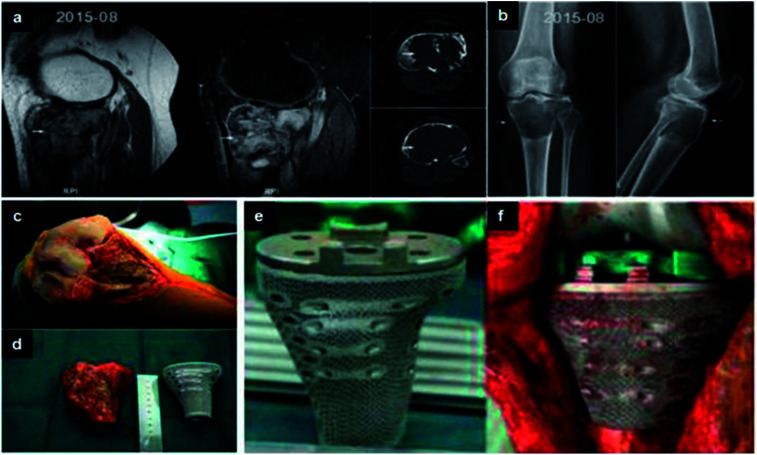
(a and b) Lesion and its boundary located in the MRI and CT. (c) The porous structure on the surface of prosthesis. (d and e) The tumor was exposed and resected. Comparing with the proximal tibia prosthesis made of titanium. (f) After tumor resection, the proximal tibia prosthesis was perfectly fitted to the bone defect. This figure has been reproduced from ref. 177 with permission from International Scientific Information, Inc.

Although conventional knee prosthesis is widely used in orthopedics, these implants would not be a good choice to surgeon in severe bone defect in distal femoral or proximal tibia.^175^ In general, the prognosis of Total Knee Arthroplasty (TKA) revision is poor due to irregular bone defect. 3DP prosthesis with high porosity is a new candidate for TKA revision. This design of tibial and femoral parts can provide micro-anchor for bone tissue. In addition, the mechanical strength was close to cancellous bone, which minimizes stress-shielding. There are good clinical results followed by 6 months.^176^ For bone tumor at the tibial plateau, the traditional hinge knee prosthesis has several problems, including stress concentration, poor motility and so on. It is difficult to replace or repair large bone defect and maintain the joint stability after the resection of tumor. Luo *et al.* produced 3D tibia block combing with standard knee prosthesis in order to treat giant cell tumor at the tibial plateau (Fig. 8). 3D microporous tibia block with customized design can fulfill the bone defect perfectly, and provide fixation points for surrounding soft tissue. In addition, the mechanical conduction of prosthesis is able to be corrected, and the retention function of the keen joint is significantly improved. The clinic result showed that the range of motion was 90°, and MSTS score was 19 followed by 7 months.^177^ Therefore, the 3DP implant can provide implant stability, retention function and soft tissue balance. 3DP prosthesis can not only repair large bone defect, but also maintain the joint stability after the resection of tumor.

### 4.4. Application of 3DP metallic implants in pelvis

With the advancements of surgical techniques and adjuvant therapy, limb-salvage surgery is a safe and efficient therapy for pelvic tumor. The anatomy of pelvic is complex, which contains important blood vessels and nervous.^178,179^ In addition, the bone defect caused by tumor is irregular and massive. Thus, it is difficult to reconstruct bone defect after pelvic tumor resection.^180^ However, the match between implant and bone defect is important to long-term stability and excellent function. Satisfactory outcomes cannot be achieved using conventional methods. AM techniques provide new possibility for the fabrication of complex shapes with porous structure, which can allow precise matching between prosthesis and bone defect.^181^ These reconstruction with high accuracy can promote long-term stability and minimize the complications. Short-term clinical outcomes demonstrate this design can achieve good clinical results without complications. Kim *et al.* implanted 3D implant into patient with sacral osteosarcoma after hemi-sacrectomy. The structure of implant includes a dense strut and porous mesh, which can provide enough mechanical support and implant stability. It was found that good bony fusion by X-ray followed by 1 year.^182^ Fan *et al.* produced 3D mirroring prosthesis for the reconstruction of pelvic chondrosarcoma. This implant can imitate the rest pelvic, and be easily implanted into the host bone. It was found that the implant had excellent alignment and stable fixation followed by 18 months.^183^ 3D sacral prosthesis can be used in recurrent sacral chordoma for one-step reconstruction after en bloc sacrectomy. The patient can walk without any pain or mechanical instability.^184^ 3D pelvic prosthesis is useful to reconstruct complex bone defect after pelvic tumor resection. In addition, the implant can achieve excellent mechanical fixation.

### 4.5. Application of 3DP metallic implants in trauma

AM can be used in the design of pre-shaped plates and production of segmental titanium truss in trauma.^185^ Traditional osteosynthesis plates should be cut/bent in order to match the intercondylar humeral fracture. However, 3D implant could be directly applied in bone defect without other treatments. Therefore, it can shorten the operative time. Shuang *et al.* implanted 3D osteosynthesis plates into patients with distal intercondylar humeral fractures, and found that the operative time is much shorter than traditional groups. There were no significant differences in the elbow function recovery followed by 10.6 months.^186^

Complex bone defect is always a tough problem in orthopedic field. When the defect size is above 6 cm, it is difficult to restructure bone defect only using bone graft. There are many cases in this field, including femoral segmental defect and tibiotalocalcaneal arthrodesis for salvaging distal tibia nonunion. It was found that 3DP implant is ideal implants when defect size exceeds 8 cm and the remaining articular surface (<2 cm) preserved in host. 3DP titanium truss cages can provide sufficient mechanical strength with the least mass, which can benefit motion and early weight bearing. In addition, these implants must be filled with bone graft for reconstruction of bone defect. In order to protect soft tissue and maintain muscle function, the design of juxta-articular implants should have a metaphyseal flare.^187^ 3DP titanium scaffold with customized design was implanted in 8.5 cm distal tibia bone defect. In order to match the bone defect, the distal of implant was designed based on the shape of the dorsal calcaneus. In addition, this scaffold with truss structure and rough surface can enhance implant osseointegration. This patient can perform daily living and walking by 6 months, and feel no pain by 15 months. CT has showed that excellent bone osseointegration between the talus and calcaneus.^188^ The case of tibial plafond fracture with nonunion was treated by 3DP titanium truss cage with a retrograde (tibiotalocalcaneal) TTC nail. For treatment of this disease, traditional implant cannot interact with host bone, which only allow for bony fusion through the central of spacer. 3DP implant was implanted into bone defect based on surgical plan, and adjusted in order to attain match between implant and bone defect. This patient can walk without other aid followed by 1 year and CT showed bony fusion in the scaffold, which would be an alternative treatment for limb-salvage surgery.^189^ All in all, 3DP segmental titanium truss is a useful implant for severe bone defects. Nowadays, many cases using 3DP implants have achieved excellent outcomes only in short-terms. Therefore, these cases should be further followed up in long-term, which is more important to value the clinical outcomes.

## 5. Directions for future potential developments

3DP metallic implant would be an optimal implant in orthopedic field. This porous structure can not only minimize stress shielding, but also promote bone in-growth for implant fixation.^190,191^ In addition, it can shorten surgical time due to its customized design. However, there are some problems in 3D metallic scaffold. (i) There is a lack of relevant laws and regulations of 3DP medical device, which would be potential risk. It is able to impede application for medical practice. Therefore, it is necessary to establish related regulatory framework and ethnic support. (ii) Many teams pursue the application of 3D implant in clinical field blindly. Due to lack of systematic understanding, surgeons only focus on customized design of prosthesis for bone defect. However, they ignore the need of treatment in clinical practice. Based on specific disease, 3DP implant should achieve the goal of vicarious function, which can significantly enhance clinical outcomes. In the future, clinical outcomes should be further followed up in long-term. (iii) It is time-consuming to apply these customized implants in patient, due to the low production efficiency. In this process, the coordinate is difficult between doctors and engineers, which can mainly impede the manufacturing efficiency. Therefore, it is necessary to enhance the efficiency of coordinate between doctors and engineers. (iv) There are many 3DP cases in recent years, due to high production efficiency. However, they are only simple repetition without intensive study. For the production of 3DP implants, it is necessary to demonstrate the clinical outcomes in intensive study. Surface modification on scaffold is a promising topic using different methods.^192^ Some techniques can promote osteoblasts adhesion, proliferation and bone in-growth.^193^ On the other hand, others can improve the anti-adhesive and antibacterial properties.^194^ Moreover, the porous structure is also an outstanding vehicle for drug delivery system. Hydrogel loaded with chemotherapy drugs can be fulfilled into scaffold, which would be used in research for bone tumor.

## 6. Conclusion

Nowadays, AM technologies open up unprecedented possibilities for producing complicated designs with customized structures, and the porous implants fabricated by AM have shown huge potential in orthopedic field. However, every AM system has its own advantages and disadvantages. It is necessary to maintain a balance between biological and mechanical properties by adjusting many factors, including the surface roughness, porosity, pore size, pore shape and pore distribution. In addition, the mechanical properties should close to bone tissue in order to avoid stress-shielding. Scaffolds with hierarchical structures would be similar to natural bone tissue. This scaffold can rebuild different types of bone tissue in different sites by AM. Furthermore, researches have proven that customized porous metallic implants can shorten surgical time and behave perfect reconstruction of bone defect. However, customized prostheses should be further followed up to assess the long-term clinical outcomes.

## Conflicts of interest

There are no conflicts to declare.

## Supplementary Material
